# Children and young people’s experiences of living with developmental coordination disorder/dyspraxia: A systematic review and meta-ethnography of qualitative research

**DOI:** 10.1371/journal.pone.0245738

**Published:** 2021-03-04

**Authors:** Áine O’Dea, Mandy Stanley, Susan Coote, Katie Robinson

**Affiliations:** 1 School of Allied Health, University of Limerick, Limerick, Ireland; 2 School of Medical and Health Sciences, Edith Cowan University, Joondalup, Western Australia, Australia; 3 Ageing Research Centre, Health Research Institute, University of Limerick, Limerick, Ireland; University of Birmingham, UNITED KINGDOM

## Abstract

**Background:**

To date services for children with Developmental Coordination Disorder (DCD) have not been informed by the perspective of children with DCD. This study aimed to synthesise the findings of discrete qualitative studies reporting the lived experiences views and preferences of children and young with DCD using a meta-ethnographic approach to develop new conceptual understandings.

**Methods:**

A systematic search of ten databases; Academic Search Complete, AMED, CINAHL, ERIC, MEDLINE, PsychArticles, PsychInfo, EMBASE, SPORTDiscus, and Web of Science, was conducted between March and April 2019, and updated in early June 2020. Meta-ethnography, following the method described by Noblit and Hare was used to synthesise included studies. The Joanna Briggs Institute Checklist was used to appraise all included papers. PROSPERO registration number CRD42019129178.

**Results:**

Fifteen studies met the inclusion criteria. Meta-ethnographic synthesis produced three themes; a) ‘It’s harder than it should be’: Navigating daily activities b) Fitting in, and c) ‘So what? I drop things’: Strategies and supports to mitigate challenges. Children with DCD describe a mismatch between their abilities and performance norms for daily activities that led to a cascade of negative consequences including negative self-appraisal, bullying and exclusion. In the face of these difficulties children described creative and successful strategies they enacted and supports they accessed including; assistance from others (parents, friends and teachers), focusing on their strengths and talents, accepting and embracing their difference, adopting a “just do it” attitude, setting personal goals, self-exclusion from some social activities, using humour or sarcasm, viewing performance expectations as a social construct, and enjoying friendships as a forum for fun, acceptance and protection against exclusion.

**Conclusion:**

Service provision for children and young people with DCD should address the social and attitudinal environments, focus on friendship and social inclusion and address stigma-based bullying particularly within the school environment. Furthermore, practitioners should identify and foster children’s own strategies for navigating daily life activities with DCD. The identified themes resonate with contemporary disability theory and the International Classification of Functioning. The social and attitudinal environmental context of children and young people with DCD profoundly influences their experiences. Future intervention development and service provision for children and young people with DCD should consider opportunities to address social and attitudinal environmental factors.

## Introduction

This neurodevelopmental disorder Developmental Coordination Disorder (DCD) affects between 5% to 6% of the paediatric population [1] and is characterised by impaired motor proficiency, which interferes with the performance of activities of daily life, academic/school-based activities, leisure and play [1]. For a diagnosis of DCD, these motor proficiency difficulties cannot be explained by other neurological conditions that affect movement [1]. DCD is associated with an array of physical health problems [2], psychosocial and mental health problems [3, 4], and learning difficulties such as reading, social skills and inattention [5]. It is well established that DCD has the potential to impact on individuals involvement and participation in daily life activities and these challenges persist beyond childhood [6].

Best practice recommendations advocate that intervention planning for children with DCD should commence with an analysis of the individual’s strengths and weaknesses in their environmental context, so that an activity or participation-oriented approach can implemented [6]. However, there is a paucity of robust empirical evidence to guide the implementation of activity or participation-oriented practice with the current available evidence hampered by a wide range of methodological issues including limited controlled trials, poor outcome measurement, and intervention design [7, 8]. Therefore, there is a pressing need to develop interventions for children and adolescents with DCD [7, 8]. It is vital that future intervention development for children and young people with DCD is informed by stakeholder perspectives in line with recommendations for complex intervention development [9].

Children with disabilities have the right to be heard on issues that affect them [10], yet research on children’s experiences has tended to ignore the views of children as active agents and ‘key informants’ in matters pertaining to their health and wellbeing [11, 12]. Similarly, there has been a lack of attention to the perspectives of children with DCD in research, service developments and policy to date. For example the recently published ’International clinical practice recommendations on the definition, diagnosis, assessment, intervention, and psychosocial aspects of developmental coordination disorder’ [6], included stakeholder representation from a parent organisation for individuals with learning disorders, but no contribution from children or young people with DCD.

Multiple researchers have emphasised the value of research on children’s experiences and perspectives where children are acknowledged as authorities on their own lives [13–15]. Historically there has been a greater focus on eliciting the perspective of parents of children with DCD rather than the perspectives of children and adolescents with DCD. A number of studies have explored parents perspectives of their child receiving a diagnosis of DCD [16], parenting a child with DCD [17–20] and parental perspectives on their child with DCD’s reduced participation patterns across activities of daily living (ADL) [21, 22], out of school activities [23], leisure-time activity participation [24], and social participation [25]. It is important to note that the perspectives of parents, educators and allied health professionals can differ from those of children with a developmental disability [26–28].

Qualitative research can illuminate the meaning of everyday experiences [29] and reveal the perspectives, views and experiences of children and young people [30]. The past fifteen years has seen a growing number of qualitative studies focusing on children and adolescent’s experiences of living with DCD [31–34], including studies on; the importance of identity and empowerment to teenagers [31], children’s perceptions of the impact of DCD on activities of daily living [32], their perceptions of participation across home and community environments [33] and quality of life [34].

Although several studies examining various aspects of the experiences of children with DCD exist, as far as we are aware, no qualitative evidence synthesis has examined the totality of research on this topic. Meta-ethnographic synthesis is a popularly employed approach to qualitative evidence synthesis because it is allows researchers to bring together multiple qualitative studies, compare accounts and develop an interpretative synthesis, which generates new conceptual understandings [35–37]. In health service research, the meta-ethnographic approach has been widely employed because of its capacity to generate new understandings on how people experience their health and well-being [37].

This study aims to synthesise the findings of discrete qualitative studies reporting the lived experiences, views and preferences of children and young people with DCD using a meta-ethnographic approach. Synthesis of the available qualitative research with children and young people with DCD will inform future research by mapping research conducted to date and has the potential to inform intervention and service delivery through generating new conceptual understandings of the experiences of this group.

## Methods

This qualitative evidence synthesis used a meta-ethnographic approach, following the seven-stage process described by [36], and the eMERGe guidance [35]. A detailed study protocol has been published previously [38] and this meta-ethnography is registered on PROSPERO, registration number CRD42019129178.

### Search strategy

The search strategy was developed from reviews examining DCD literature [39] and qualitative research [40]. A combination of keywords, thesaurus and MeSH terms were utilised (an example of the search strategy used in MEDLINE is presented in S1 File. Ten databases were searched; Academic Search Complete, AMED, ERIC, CINAHL, MEDLINE, PsychArticles, PsychInfo, EMBASE, SPORTDiscus, and Web of Science between March and April 2019. In the first week of June 2020, the search strategy was administered across the 10 databases in order to identify any further articles that may have been published between April 2019 and June 2020. Searches were limited to English language publications, but no limits were applied to the date of publication. The PRISMA-checklist for systematic reviews was used to illustrate the search strategy procedures[41], which is presented in S2 File.

### Inclusion & exclusion criteria

Primary research studies using qualitative methods of data collection and analysis to explore children and young people’s (5–18 years) views, opinions, and experiences of living with DCD, were included. Full details of the inclusion and exclusion criteria are presented in S3 File. Studies were excluded if (a) they included participants with a range of neurodevelopmental diagnoses and the qualitative data for those with DCD could not be extracted, or (b) the data presented was aggregated (for example, a mix of parent and child data that cannot be easily identifiable).

### Screening

**Fig 1:**
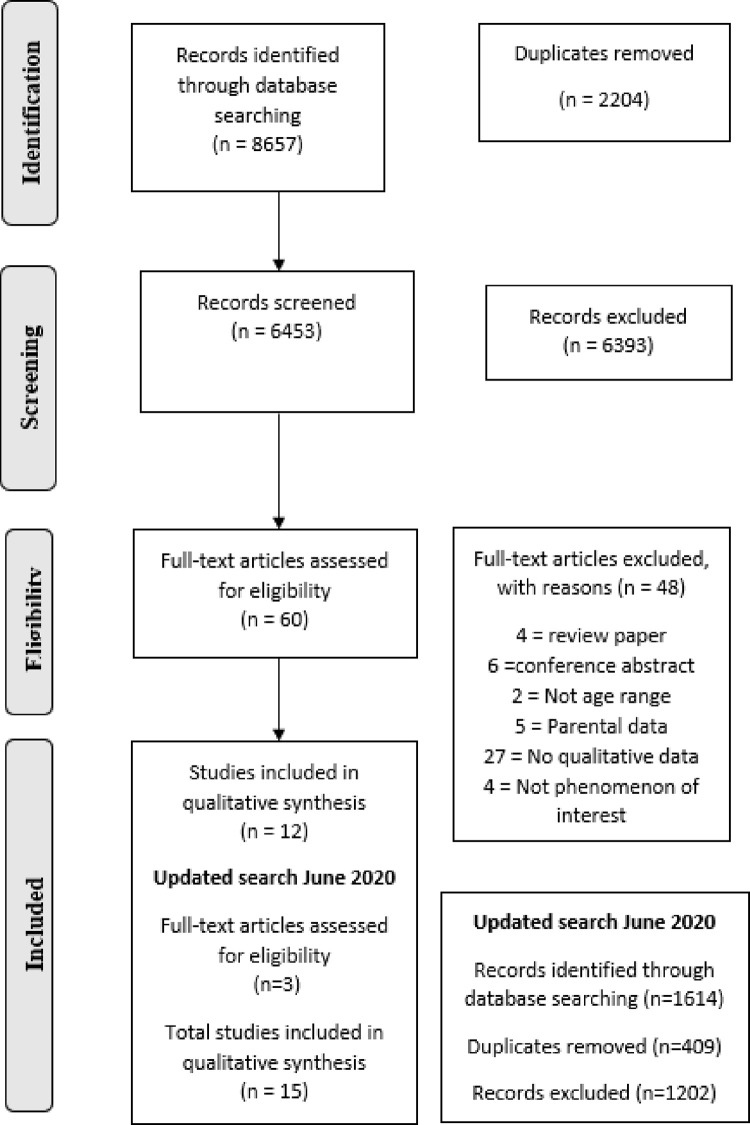
PRISMA flowchart.

Once duplicates were removed, the first author (ÁOD) screened papers by title and abstract against the pre-designed inclusion/exclusion criteria (S3 File). KR screened 10% of papers by title and abstract to check for consistency. Papers included for full-text review were read and screened by ÁOD and KR. Each reviewer independently considered the paper’s relevance to this qualitative synthesis, any differences of opinion were resolved via discussion. The entire screening process is presented via a PRISMA flowchart in (Fig 1).

### Data extraction and analysis

This study followed the analytic and synthesis phases of meta-ethnography outlined by Noblit and Hare [36], and the eMERGe guidance [35]. The raw data of meta-ethnography comprises participants’ views, explanations or perceptions of a phenomena in original studies, and the study authors’ interpretations and conceptualisations of these data [42]. Participants’ descriptions of their experience of the phenomena, (in this case; living with DCD), are labelled first-order constructs [42]. The researchers’ analysis and interpretation of these first-order constructs are labelled as the second-order constructs [42]. Third-order constructs are the synthesis of the researchers’ interpretations of the second-order constructs [42]. Noblit and Hare detail the importance of constructing ‘adequate interpretative explanations’ through the selection of key metaphors or concepts while preserving the sense of original accounts (pg.13 36). Metaphors are “what others might call themes, perspectives, organizers and /or concepts revealed by qualitative studies” (pg.15 36). The full-text pdfs of included papers were uploaded to QSR International’s Nvivo 12 software, so that first-order and second-order constructs could be extracted and inductively interpreted to identify the key concepts/metaphors. This software platform provided a useful tool to support the documentation of interpretative concepts generated as the data were repeatedly read and interpreted [38].

Simultaneously, two independent reviewers used a data extraction tool designed specifically for this study to extract and collate information on the characteristics of each study, including; citation, study setting/country, sample size, participant characteristics, aims of the study, data collection and methods, and summary of findings. The extracted data were tabulated to support a broad overview of the included studies [43]. During the synthesis process, the researchers returned to reading and re-reading the full text papers to facilitate immersion in the data and to enhance the analytical rigour and synthesis [35, 36, 43]. To support analytic rigour, one conceptually rich study [44] and one study identified to have some methodological limitations [45] were intentionally selected, read, and independently interpreted by two authors (ÁOD & KR). The interpretative concepts generated by both researchers were discussed until agreement was reached. A similar process was applied to the remaining papers by ÁOD, whereby, interpretative concepts were generated from the extracted data. The research team convened several meetings to discuss these third-order concepts and similarities and differences between the concepts, in order to translate the studies into one another [36, 42]. ‘Translation’ of studies in meta-ethnography is described by Noblit and Hare as a process where studies are treated as analogies and compared with one another [36]. This method of constant comparison of the data identified that the key concepts across the studies were similar and could be added together, thus allowing the researchers to reciprocally translate the studies into one another [36, 42]. During meta-ethnography when the relationship between studies is considered researchers, decide if the synthesis is a refutational synthesis (differing accounts) or a reciprocal synthesis (accounts being similar) [42, 43]. Although there was a difference in the age ranges across the included studies, the accounts were not in opposition to each other. The variance in age ranges from childhood to adolescence helped to support the interpretative explanations developed in the synthesis [36]. Through discussions, these third-order constructs were further refined and developed. The final stages phase involved the research team synthesising the third-order constructs into a line-of-argument, which provides greater conceptual understanding to the phenomena of interest as a whole; that is children and young people’s perspectives and experiences of everyday life and living with DCD.

The researchers identify their philosophical position as interpretivist, this position aligns with the aim of the study and the assumptions underpinning meta-ethnography. Noblit and Hare [36] highlighted that a meta-ethnographic synthesis exposes much about the perspective of the synthesizer as it does about the body of the synthesis. For that reason, the first author who is an occupational therapist with an extensive clinical background in working with children and young people with DCD in paediatric services was cognisant of the potential influence of prior experience on her interpretations. However, the researchers (KR, MS & SC) do not share this background and have extensive qualitative research experience. Throughout, the study reflective discussions within the research team challenged the first author’s interpretations and meanings of conceptual concepts. However, ultimately, the authors acknowledge that our interpretations of the key concepts and subsequent reciprocal translations are only one potential reading of the studies [36].

### Quality appraisal of the included studies

The methodological congruency of the included papers was appraised using the JBI Checklist [46]. Two reviewers (ÁOD and KR) independently appraised each paper. The JBI checklist is deemed to be one of the most sensitive tools when examining methodological validity, given its focus on congruity [47]. Each item was recorded as “Yes”, “No”, “Unclear” or” Not applicable”. Once complete, the appraisal findings were contrasted, variations in decisions were examined and consensus was reached via discussion between ÁOD and KR. In addition, quality appraisal decisions were discussed amongst the wider research team. Toye and colleagues [48] acknowledge that current quality appraisal checklists can produce inconsistent decisions. The research team discussions focused not just on methods alone but on the contribution of individual papers and the strength of its concepts to facilitate the generation of interpretative concepts and subsequent conceptual insight to the meta-ethnography [48]. For this reason, the researchers prioritised the discussion of decisions to facilitate consensus.

## Results

### Study selection

Initial searches yielded 8657 results, 6453 after removing duplicates. Screening by title and abstract excluded 6393 results, leaving 60 studies for full text review. Twelve studies met the inclusion criteria at this stage (March 2019). In the first week of June 2020 the search was updated 1614 articles were identified, after removing duplicates 1205 papers were screened. A further three papers were included. Fig 1 presents a PRISMA Flowchart diagram, detailing the entire process, which led to the inclusion of 15 studies.

### Study characteristics

The characteristics of the fifteen included papers are presented in Table 1. Six of the studies were conducted in the United Kingdom [31, 45, 49–52], five in Canada [33, 34, 44, 53, 54], one in Austria/ Italy [55], one in Brazil [56], one in Belgium [57], and one in New Zealand [58]. In total, data from one hundred and nine participants were included in the qualitative synthesis. Nine papers had participants with a mean age ranging from 6.9 years to 11 years [33, 34, 45, 49, 54–58]. Five papers had participants with a mean age range of 13 years to 14.9 years [31, 50–53] and one study included young adults aged 19–25 years who reflected upon their experiences as an adolescent [44]. Two papers included data from one sample of young people interviewed over a two-year period [51, 52].

**Table 1:** Characteristics of included studies.

Author(s) and year Country	Sample characteristics	Research aim	Methodology & analysis	Data collection	Summary of findings
Armitage, Swallow and Kolehmainen (2017) United Kingdom	Children aged 7–11 years Total study sample n = 7 Data extracted for children with DCD (n = 3)	(1) To explore children with coordination difficulties views of the key ingredients of occupational therapy interventions. (2) To examine the processes through which these ingredients might relate to the children’s participation outcomes.	Grounded theory methodology	Individual interviews or interviewed with a parent present. Picture cards, drawings and common therapy materials such as gym balls, scooter boards and pencil grips were used as prompts when interviewing children.	The study identified intervention ingredients: performing activities and tasks, achieving as conceptual categories of ingredients, which support children’s participation.
Barnett, Dawes and Wilmut (2013) United Kingdom	Boys aged between 13–15 years Study sample n = 8	To understand which factors constrain and facilitate participation in physical activity for teenage boys with DCD.	Qualitative, categorical-content analysis	Individual semi-structured interviews with the children. Interview duration ranged between 13–20 min for children.	Two main themes, internal and external factors were constraints and or facilitators to physical activity. Internal constraints fell into three main sub-themes: motor skill and confidence, poor motivation and lack of time, fatigue and pain. External constraints and facilitators included both physical and social factors, falling into five sub-themes: facilities and transport, peers, family, teachers, instructors and activities/tasks.
Costa, Brauchle and Kennedy-Behr (2017) Austria & Northern Italy	Children aged 5–10 years Total study sample (n = 34) Data extracted for participants with a diagnosis of DCD (n = 9)	To explore collaborative goal setting with children, parents, and teachers, and children’s reasons for their goals based on their perceived self-efficacy, using the Austrian–German Perceived Efficacy and Goal Setting System (AG-PEGS).	Exploratory mixed methods Content analysis	The Austrian–German Perceived Efficacy and Goal Setting System (AG-PEGS) was administered as a semi-structured interview and the child was asked for his/her reasons for choosing particular goals.	Children explained their reasons for their selected goals, which could be grouped into three major categories: social motives; their independence (mainly related to self-care tasks with impact on social participation), and ease, competence and joy when engaging in a certain activity.
Coussens *et al*. (2020) Belgium	Children aged 5–9 years Total study (n = 16) Data extracted for participants with a diagnosis of DCD (n = 8)	The main aim of this study is to investigate how young children with DCD experience participation.	Qualitative research design using a photo elicitation approach Thematic analysis	In-depth interview with the child based on their photographs	Four main themes including, playing together, learning and family gatherings, and barriers and facilitators to participation. Children perceived their participation as satisfying when they can play, learn and join in family gatherings resulting in feelings of inclusion, recognition and belonging.
Dewey and Volkovinskaia (2018) Canada	Young people aged 11 to 18 years. Total study (n = 44) n = 24 participated in qualitative interviews	The aim of this study was to acquire a better understanding of Health Related Quality of Life and peer relationships in adolescents with DCD and ADHD.	Mixed methods Content analysis	Individual semi-structured interview	The responses of the adolescents were categorized into three broad categories: leisure time/school activities, friendship, and popularity/peer victimization.
de Medeiros *et al*. (2019) Brazil	Children aged 7–10 years (1 boy; 2 girls) Study sample (n = 3)	This study aimed to investigate the perceptions of the child about the likely impacts of DCD on children’s occupational performance and family daily life.	Exploratory qualitative approach Content analysis	A free-drawing activity was proposed to the child, while the researcher conducted an open interview	The main themes included opportunities to play and do, and relationships with others.
Foulder-Hughes and Prior (2014) United Kingdom	Children aged 10–11 years. (5 boys, 1 girl) Data extracted for participants with a diagnosis of DCD n = 3.	To investigate how children felt about the transition from primary to secondary school.	Exploratory qualitative approach Thematic analysis	Individual semi-structured interviews with children	The main themes that emerged from the analysis were worries and support strategies.
Hessell, Hocking and Davies (2010) New Zealand	Children aged 7 or 8 years Study sample (n = 3)	To explore whether children with DCD could effectively participate in a community gymnastics group and what internal and external factors influence successful participation. To provide a detailed description of why gymnastics is accessible to children with DCD.	Ethnography	Data gathered through observations and written up in the form of field notes. In-depth semi structured interviews were conducted with the three gymnasts with DCD after 18-week block.	Findings revealed that influences from the environment beyond the Club informed the gymnastic culture in which gymnasts with DCD participated. Graded skill development, variation of activity, and individual measures of success supported their involvement.
Jasmin *et al*. (2018) Canada	Children and young people aged 6 to 13 years (mean age = 8.9 SD:2.6) (8 boys; 2 girls) Study sample (n = 10)	To explore children’s views and perceptions of their participation needs at home and in the community.	Multiple case studies Qualitative approach Thematic analysis	Individual semi-structured interviews	Two main categories 1. Participation at home—children’s interests; challenges and expectations regarding children’s participation at home; support or services received and requested at home. 2. Participation in the community—Children’s interests; challenges and expectations regarding children’s participation in the community; support or services received and requested in the community.
Lingam *et al*. (2014) United Kingdom	Young people aged 11–16 years Study sample (n = 11) (7 boys, 4 girls)	To gain an in-depth understanding of the experiences and aspirations of a group of young people, living in the UK, diagnosed with DCD.	Phenomenological hermeneutic method	Individual face-to-face semi-structured interview and a subsequent further group interview to expand on the points made within the initial interviews.	The central theme of ‘We’re all different’ described how the young people saw themselves. This concept incorporates five subthemes: ‘How I see my life’, ‘Things I find hard’, ‘Making my life easier’, ‘How others see me’ and ‘How I see my future’.
Missiuna *et al*. (2008) Canada	Young adults aged 19–26 years (mean 21.5 years) Study sample (n = 9) (4 Male, 5 female)	This project aimed to explore the effects of coordination difficulties on the life domains of adolescence.	Phenomenological approach Qualitative analysis	Two in-depth interviews were conducted with each participant. A follow-up interview focused on specific issues that emerged from the initial interview and from the questionnaires, including perceptions of changes that occurred over time plus key barriers and supports during adolescence.	Three main themes. The first relates to understanding coordination differences in context; the second relates to strategies that the participants used to manage their differences; and the third theme relates to how the differences evolved over time.
Payne *et al*. (2013) United Kingdom	Young people aged 13 years Study sample (n = 6) (5 boys, 1 girl)	To investigate the experience of teenagers living with DCD, from their perspective.	Interpretative phenomenological analysis (IPA)	Individual semi-structured interview ranged from 40–60 minutes.	This study presented one theme: the social impact of living with the condition discussed in this paper.
Payne and Ward (2020) United Kingdom	Young people aged 13–15 years Study sample (n = 9)	The aim of the study was to gain a further understanding of the lived experiences of teenagers with DCD from their own contemporaneous perspective.	Interpretative phenomenological analysis (IPA)	16 interviews were carried out with nine teenagers over a 2-year period.	Study themes included were: ‘Doing everything the hard way’; ‘I didn’t want to be seen as anyone different’; ‘I’m an intelligent person but I can’t even write’; ‘Right help, right time’ and ‘Making sense of the diagnosis’. Self-efficacy was a strong recurring theme, influencing participants’ motivation for and participation in daily activities, and affecting teenagers’ sense of resilience, agency, ambition and identity.
Spencer-Cavaliere and Watkinson (2010) Canada	Child aged 10 years Total study (n = 11) Data extracted for 1 male participant with DCD	To examine inclusion from the perspectives of children with disabilities in physical activity.	Exploratory qualitative approach Content analysis	Individual interviews approximately 30 min.	Having friends was identified as a theme. This theme was associated with feeling included was associated with play.
Zwicker *et al*. (2018) Canada	Children aged 8–12 years (Mean age 9.9yrs) Study sample n = 13 (10 boys, 3 girls)	To examine the implications of DCD on the daily life of children and deepen the understanding about Quality of Life of children with DCD.	Qualitative approach incorporating the use of photo elicitation Thematic analysis	Individual semi-structured interviews 25 to 80 minutes.	Four interrelated themes provide insight about the experience of living with DCD, from participants’ perspectives: (1) milestones as millstones: struggling to perform ordinary activities; (2) the perils of printing: schooling as hard work; (3) more than a motor problem: left out of left field; and (4) coping strategies: thinking differently and emphasizing strengths.

### Quality appraisal

Appraisal of the included papers highlighted a large variation in methodological quality across the studies as seen in Table 2. Ten studies presented the philosophical perspective on which the study was based and a methodological approach which was congruent with the perspective [31, 34, 44, 51, 52, 54–58]. However, the philosophical or theoretical perspective was not clearly represented in the remaining five papers [33, 45, 49, 50, 53]. The majority of studies described an appropriate qualitative methodology for addressing the research question or objectives. Two areas rarely addressed were the researcher’s cultural and theoretical orientation, or the influence of the researcher on the research and vice versa. The reporting of methodological quality was deemed unclear across many items in one study [45]. However, we chose to include the paper, as we believed that the representation of participants and their experiences was clear. Across all studies, participants were clearly represented from the data presented in the findings. One study was included even though it did not include a statement about ethical approval; however, the authors did describe the process for gaining child assent and parental consent [55]. No studies were excluded based on quality.

**Table 2:** JBI appraisal checklist for qualitative research (rated as yes, no, unclear).

Citation	Question 1	Question 2	Question 3	Question 4	Question 5	Question 6	Question 7	Question 8	Question 9	Question 10
	Is there congruity between the stated philosophical perspective and the research methodology?	Is there congruity between the research methodology and the research question or objective?	Is there congruity between the research methodology and the methods used to collect data?	Is there congruity between the research methodology and the representation and analysis of data?	Is there congruity between the research methodology and the interpretation of results?	Is there a statement locating the researcher culturally or theoretically?	Is the influence of the researcher on the research, and vice-versa, addressed?	Are participants, and their voices, adequately represented?	Is the research ethical according to current criteria or, for recent studies, and is there evidence of ethical approval by an appropriate body?	Do the conclusions drawn in the research report flow from the analysis, or interpretation, of the data?
[49]	Unclear	Yes	Yes	Yes	Yes	No	No	Yes	Yes	Yes
[50]	Unclear	Yes	Yes	Yes	Yes	No	No	Yes	Yes	Yes
[55]	Yes	Yes	Yes	Yes	Yes	No	No	Yes	Unclear	yes
[57]	Yes	Yes	Yes	Yes	Yes	Yes	Yes	Yes	Yes	Yes
[53]	No	*Yes*	*Yes*	*Yes*	*Yes*	No	No	Yes	Yes	Yes
[56]	Yes	Yes	Yes	Yes	Yes	Yes	No	Yes	Yes	Yes
[45]	Unclear	Unclear	Unclear	Unclear	Unclear	No	No	Yes	Yes	Yes
[58]	Yes	Yes	Yes	Yes.	Yes	Yes	Yes	Yes	Yes	Yes
[33]	Unclear	Yes	Yes	Yes	Yes	Yes	No	Yes	Yes	Yes
[31]	Yes	Yes	Yes	Yes	Yes	No	No	Yes	Yes	Yes
[44]	Yes	Yes	Yes	Yes	Yes	No	Unclear	Yes	Yes	Yes
[52]	Yes	Yes	Yes	Yes	Yes	No	No	Yes	Yes	Yes
[51]	Yes	Yes	Yes	Yes	Yes	No	Unclear	Yes	Yes	Yes
[54]	Yes	Yes	Yes	Yes	Yes	Yes	No	Yes	Yes	Yes
[34]	Yes	Yes	Yes	Yes	Yes	Yes	Yes	Yes	Yes	Yes

### Synthesis

This meta-ethnographic synthesis of first and second-order constructs produced three interrelated themes (third order constructs); a) ‘It’s harder than it should be’: Navigating daily activities b) Fitting in, and c) ‘So what? I drop things’: Strategies and supports to mitigate challenges.

#### ‘It’s harder than it should be’: Navigating daily activities

This theme relates to the difficulties children and young people with DCD face performing everyday activities. Children and young people describe the influence of personal factors (such as self-perceptions, motivation, and age) and environmental factors (such as family and school context and support) on their capacity to navigate and perform daily life activities.

The experience of struggling to learn and perform everyday activities and routines was frequently reported across the included studies [31, 33, 34, 44, 45, 49, 51, 52, 55, 57]. Typical childhood activities such as learning to ride a bicycle required additional support and extended periods of practice to master skills and build stamina [33, 34, 44, 49, 52]. In late adolescence, different activity challenges arose such as learning to drive [44, 51].

Children and young people evaluated their performance and functional abilities in everyday scenarios against siblings and peer’s performance, and parental expectations [31, 33, 34, 44, 50, 52, 53, 55, 56]. Self-appraisal was often critical when performance did not align with context expectations [31, 34, 44, 50, 52, 53]. Participants used words like “stupid clumsiness and awkward” to describe themselves and their motor difficulties [44]. Challenging performance in everyday activities contributed to a sense of personal inadequacy for some participants [34, 44, 50, 52, 55]. Negative self-perceptions and fear of exposing their performance difficulties influenced participants’ engagement and willingness to try social and physical activities [34, 44, 50, 52]. As articulated by this young boy: ‘‘I always think I’m a loser and um, you know feel kind of sad for quite a long time but I’ll get over it. It’s really sad and you don’t think you can do it. And you stop trying” [34]. These perceptions left some children and young people feeling different from their peers and siblings [31, 34, 44, 52, 53] as illustrated in this young person’s description:

I couldn’t write properly, I couldn’t play sports properly, and I was always spilling things or breaking things. It just makes me feel so—like you’re so different from other people, and nobody can ever really understand. It would make me feel like there was something wrong with me personally, when really I couldn’t really help it [44].

Requiring parental assistance to complete or accomplish activities left some children feeling embarrassed and guilty, and perceiving themselves as a burden on their parents, because they could not perform activities independently or without creating a mess [34, 44, 50, 52]. However, in most cases children and young people recognised their need for parental assistance and perceived assistance positively [31, 33, 34, 44, 50, 52, 57, 58]. Participants spoke frankly about parental support, which was provided in various ways, such as teaching skills, scaffolding the demands of the task, helping the child understand their difficulties [34, 52, 57], advocating for academic support [31, 52], and providing practical and emotional support that facilitated participation in community and leisure activities [50, 57, 58].

My mum and dad on a Sunday go to the gym with me… they encourage me to do more like stuff in the gym… If they didn’t go to the gym with me than I probably wouldn’t do any physical activity at all [50].

Motor performance difficulties impacted on school life [31, 34, 44, 45, 50–53] where some perceived that they were not performing adequately and were at the lower end of achievement for their grade [31, 34, 51, 55]. Specific school-based activities that were challenging included handwriting, self-management of learning, physical activity, sports, and recess time.

Physical education classes, school sports, recess time, and out of school activity were associated with performance difficulties and emotional distress [31, 34, 44, 45, 50, 52, 53]. Factors such as fatigue, pain, poor performance endurance and fear of injury influenced participant’s motivation, involvement, and enjoyment of physical activities in school activities and out-of-school activities [33, 34, 44, 50, 57, 58]. As summed up by this participant “It’s just hard to get going, hard to keep going” [50].

Handwriting was experienced as hard work and a significant problem that affected participation in school [31, 34, 44, 51, 52], which in some cases warranted intervention [31, 33, 34, 49, 51, 52]. Handwriting difficulties were pervasive and included legibility, letter formation, speed of performance, fatigue, and pain [31, 34, 49, 51, 52]. Participants received support via targeted handwriting practice, use of computers and tablets, typing programs and personnel-based support such as scribes and Learning Support Assistants [31, 34, 44, 51, 52]. However, access to learning supports was not equal; in one study participants from lower socio-economic backgrounds were promised additional help and resources but these did not materialise [31]. Young people expressed relief when the environmental context changed and writing was no longer a priority [44]. In contrast, children and young people perceived generic strategies as unhelpful or ineffective when imposed without considering their individual needs [34, 51].

I’ve done three, two, typing programs both, the whole thing at my home and I’ve done the same typing program almost twice in school and it’s still, I’m still looking down and I can’t really type fast [34].

The perceptions of others, including teachers, peers, parents, and siblings influenced participant’s perceptions of their handwriting, and scholastic success [31, 34, 44, 51, 52].

If someone said something to me, like a teacher about handwriting or something. I would just be less confident for the rest of the day [51].

The teachers’ level of understanding of handwriting difficulties varied with some teachers compounding children’s difficulties, as illustrated by the quote.

It’s harder than it should be. And then most of my teachers except for one were stupid and like, didn’t notice… I lost a lot of marks in school because I had messy handwriting which I didn’t think was fair… They said oh, I’m just lazy… So how would they feel if someone called them lazy when they’re working their hardest. Or when you make a mistake and they point it out to you every waking moment [34].

Other participants reflected negatively upon their handwriting and scholastic performance compared to their siblings and peers [31, 34, 52, 56]. As highlighted by this adolescent boy, “My sister’s got better writing than me … Don’t get me wrong, I’m an intelligent person, but like I can’t even write. It’s making me fill up” [52]. In contrast, other participants reported that they were doing well in school and valued the positive reinforcement that they received from teachers, parents and siblings [31, 34, 51]. Extra special attention was not wanted [31]; rather participants appreciated help when teachers and learning support staff demonstrated an understanding and awareness of their needs [31, 34, 51].

Challenges with executive functioning skills and self-management affected participants’ independence in school, during class-based learning experiences, and out of school activities. Children and young people detailed difficulties with the planning, organisation and remembering personal belongings, concentration, and memory skills [31, 34, 51, 52, 55]. Participants were aware of the challenges that they faced understanding and processing new information in-class [31]. As described by this participant; ‘It is not because he or she is going fast it is just me not knowing because I don’t understand what they mean’ [31, 52]. In contrast, some participants’ used supportive strategies, such as sitting beside a friend or a Learning support assistant so that they could explain the information again [57]. While, others felt that an individualised learning approach suited their ability to learn new skills and information [31, 44, 52].

#### ‘Fitting in’

This theme relates to children and young people with DCD’s desire to participate and to be socially included in peer interactions and experiences in everyday life situations. Friendships were desired, and positively experienced however, experiences of marginalisation; exclusion and bullying because of the mismatch between their skills and performance expectations were common. Across the included studies, friendships were reported as desired [31, 34, 44, 45, 52–55, 57], and provide a forum for fun, acceptance of individual difference, and shared interests [31, 52, 53, 57]. Children with DCD describe pleasure in playing with others, as it facilitates a sense of ‘inclusion’, ‘companionship’ and ‘friendship’ [57]. In the school context, the protective nature of friendships against social exclusion, being teased or bullied was deemed particularly important [31, 52–54]. Friends were viewed, as “one of the best things about school” [31], and the opportunity to hang out with friends outside school was highly regarded [31, 52]. Friendships helped children and young people to evaluate and identify positive self-perceptions [31, 51, 52].

A lot of my mates say I am funny; I think I am quite funny and I am quite strong and um, my mates have told me I am quite reliable [31].

Having additional needs or being perceived as different from their peers was the impetus that led some young people to forming social connections, but such connections also helped participants to reframe their perceptions about difference [31, 52, 53].

They’ve [his group of friends] got like all different like talents, if you get my drift like…but most are into different sports and that [31].

Children transitioning from primary to secondary school worried about making new friends, [45]. Some noted that with transition to high school "that certain people made friends just because they were on a sports team" [44], reflecting the premise that participation in physical activities provided increased social opportunities [44].

Social and attitudinal environments influenced acceptance and the opportunity to participate in activities. Children with DCD commonly experienced challenging social situations, particularly with peers, ranging from marginalisation and exclusion to bullying [31, 34, 44, 45, 50, 52–56]. Exclusion typically occurred in the school context [31, 34, 44, 45, 50, 52, 53, 56]. Children and young people perceived that the most noteworthy barriers that led to exclusion or being left out from participating in specific groups or activities was the ‘mismatch’ between the performance standard and their ‘skills’ and ‘competences’ [34, 44, 50, 52, 53, 57]. As described by this young person, “I tried to help earlier but I wrecked everything, so he won’t let me do anything that involves making characters” [52].

Bullying was encountered or feared in the school setting by many [31, 34, 44, 45, 50, 52, 53]. Participants articulated experiences of negative name-calling, being laughed at, and being teased or ridiculed about their motor performance [31, 34, 44, 45, 50, 52, 53, 56].

Everybody laughs at me when I try to run on the grass… They sometimes bully me and chase me and all that [34].

Being teased or ridiculed also occurred in the home setting between siblings, as described by this participant, her brother had told her that ‘she was going to be a prostitute when she grew older as she did not like school’ [31]. Reported incidents of physical harassment and assault, were traumatic and left the individuals feeling angry, vulnerable, and unsafe [31, 34, 52].

Sometimes I don’t even want to go to class… it was fine when people knew [about the diagnosis] and just said stuff, but now they’re going on like about stabbing people [31].

Participation in school sports activities was often associated with distressing experiences of marginalisation and bullying [34, 44, 50, 52, 53]. Negative self-perceptions result from this mismatch between performance and expectations.

Teacher awareness also influenced the experience of school sport,

I have had some really bad experiences with PE… the teacher can get frustrated quite easily, ‘they were like screaming at me and saying ‘run properly boy’… I just don’t like to be yelled at to do stuff [50].

Teacher awareness and attitude towards bullying was considered important [31, 52], but children and young people did not typically perceive teachers as a helpful resource to dealing with bullying [45, 52]. Marginalisation and social exclusion were associated with feelings of loneliness, sadness, and frustration [34, 53], and a wide range of negative self-perceptions [31, 34, 44, 52, 53]. Young people perceived that they were viewed as different, as reported by this 14-year-old girl, “they just think that we’re weird” [53].

#### ‘So what? I drop things’: Strategies and supports to mitigate challenges

Although children and young people with DCD describe numerous challenges and difficult experiences in everyday life, embedded within these descriptions were multiple supports and strategies used to mitigate challenges and facilitate participation. Personal factors such as the child and young person with DCD’s preferences towards activities and life situations, their sense of self, age, and insight into their health condition influenced their capacity to join in and be involved.

Across the studies, many participants spoke positively about their everyday lives. They experienced enjoyment in family, play and leisure-based activities, which they chose to, participate in, and performed competently [31, 33, 34, 44, 49, 50, 52, 53, 56–58]. As described by this child’s preferred activities in school and the community context; “Hide and Seek… Chess, we play dolls at school… Soccer, I like dodgeball too”[56]. Participants focused upon their interests, strengths and what they could do, or a unique attribute and personal quality [31, 34, 44, 52].

There is always something that you can do that other kids can’t…. Like your talent, everyone has a talent [34].

Although some participants were unsure of how they would manage in the future, for others, the challenges they faced did not limit their future goals and aspirations such as learning to drive or going to college or university [31, 52].

Another common strategy was adopting a “just do it” attitude [31, 34, 44, 51, 52]. Participants’ descriptions revealed a growth mindset, towards practice and perseverance of skills/activities, that they had “not yet” mastered [31, 34, 44, 52]. With age, teenagers reflected that they had developed the competencies to perform most basic self-care activities, “not like perfect, but to a standard that’s OK” [51]. Participants’ interests and motivation to join a peer play or recreational activity influenced their desire to persist with and practice challenging activities [31, 34, 44, 49, 52, 55]. Equally, as children and young people mature, they recognise and accept the challenges they experience, which in turn helps them to develop autonomy and confidence to manage everyday life scenarios [31, 34, 44, 51, 52].

What I was good at, I kept doing, and what I wasn’t good at, and I stopped doing. Then I started being more confident in everything I did because everything I did I could do [44].

Understanding the diagnosis of DCD influenced participants sense of self that is it helped young people’s self-confidence and self-perceptions [31, 34, 44, 49, 51, 52] and allowed participants to accept and embrace their difference [31, 51].

Knowing that I’ve got it. That I’m not just a bit weird …. I’m not ashamed of it because it kind of makes me who I am. I think that everyone has their problems [31].

Therapeutic intervention helped others to understand their difficulties; in turn, this enhanced their confidence, and perception that they were not the only person to struggle with everyday activities [31, 49]. Role models such as the actor Daniel Radcliffe who identifies as having DCD helped others to associate with a positive identify [51]. The perception that DCD was a hidden disability was present “nobody ever really understand…it would make me feel like there was something wrong with me personally” [44]. However, a more common perspective was that performance and context expectations were a social construct, and therefore participants did not internalise their experience of everyday activities [31, 34, 44, 51, 52].

When I look at it, it’s not a problem… . I never felt like it was me as much as it was something imposed on me by other people because they were the ones who were criticizing or passing comments… . So what? I drop things. It doesn’t matter. It only becomes a problem when you think that dropping things isn’t normal [44].

Children valued the opportunity to set personal goals for skills/activities that they wanted to develop and practice [34, 49, 55]. Goals were realised through therapy for some children [49, 55], while others engaged in extensive practice with a parent [34], where tailored practice enhanced children’s confidence and self-perceptions [34, 49]. The sense of achievement and involvement when children mastered cycling was clear: “I’m better at riding a bike now…we [family]was in Yorkshire…I was OK on this bike ride…it [therapy sessions] just makes me think.. ‘I can do it’ so I won’t have that much problem pedalling [49].

Children practiced adaptive self-exclusion from social everyday activities that were not inclusive, potentially exposed their difficulties, or where they lacked the self-belief that they could participate successfully [34, 44, 50–52, 56–58]. For example, a 13-year old with DCD reported *“*If it was a team sport with people that I didn’t know particularly well, then I wouldn’t particularly want to get involved in case I got it wrong” [52]. Whilst self-exclusion was a common strategy for many children this strategy was not without its drawbacks. As highlighted by some young peoples’ reflections, who wished that, they had persevered for longer with activities, as, this strategy had limited subsequent participation opportunities as an adult [44].

“Now that I’m older, I kind of wish I had gotten into things earlier, because now I wish I was involved in some sort of group activity, sport, something, you know? My friends now, some of them are cheerleaders, and some of them [play] lacrosse or whatever, and I still don’t have anything” [44].

Many children and young people with DCD described the use of humour or sarcasm as a strategy to support inclusion [34, 44, 53]. As revealed by this 12-year-old boy with DCD: “Mostly, I just go around collecting the dodge balls and say “dodge balls! Need ammo? Here!” [53]. Learning to laugh at themselves and joke about their activity performance were viewed as means to boost involvement in an activity, reduce frustration, and deflect negative peer attention [34, 44, 53].

## Line of argument synthesis

The results of the 15 papers were reciprocally translatable and led the development of three interpretative themes. Higher order interpretation of these themes identified that the experiences of children and young people with DCD can be understood as the experience of psycho-emotional disablism, a concept developed within the field of disability studies. Carol Thomas [59] conceptualises psycho-emotional disablism as occurring when individuals with impairments are ‘hurt by the reactions and behaviours of those around them, made to feel worthless, of lesser value, unattractive, hopeless, stressed or insecure’.

Across the included studies, children with DCD described struggling to meet their parents, teachers, peers and broader society expectations around performance of activities. The mismatch between their abilities and performance norms in many cases led to a cascade of negative consequences including self-criticism, negative self-appraisal, bullying, victimisation, marginalisation and exclusion by peers and siblings. Across the included studies numerous examples of psycho-emotional disablism arising from relationships with other people, were reported, for example critical comments such as teasing or actions such as exclusion. Another manifestation of psycho-emotional disablism, internalised oppression, was also evident in self-criticism and negative self-perceptions. Further underscoring the significance of the social and attitudinal environment on the experience of children with DCD, those who encountered positive and encouraging social and attitudinal environments across a range of environmental contexts navigated situations with greater ease and detailed experiences that are more positive. In response to disablism, children and young people developed numerous strategies and drew on available supports to facilitate their involvement in activities and enhance social inclusion across life situations. Children with DCD revealed that they accessed support to diminish the mismatch between their performance and the expectations of others, including seeking and accepting assistance, focusing on their strengths and talents, accepting, and embracing their difference, adopting a “just do it” attitude, setting personal goals, self-exclusion from some social activities, using humour or sarcasm, and viewing performance and context expectations as a social construct.

## Discussion

This synthesis reveals important insights into the lived experiences of children and young people with DCD. Three interrelated themes were identified and labelled, as ‘It is harder than it should be’: Navigating daily, Fitting in, ‘So What? I drop things’. Across the studies in this review, participants experienced a wide array of challenges learning and performing new skills and activities, participating in school, and experienced social exclusion from activities such as play and leisure. This aligns with much of the quantitative research to date on DCD in childhood, which has focused upon the predictors of patterns of participation [60]. However, this qualitative review focused upon the lived experience of children with DCD and exposes the array of self-developed strategies and successfully accessed supports used by participants to deal with these challenges and navigate everyday life situations. Findings of this synthesis resonate with the social model of disability and the ICF.

Considering the synthesis findings in the context of the social model of disability and the ICF suggests that the social, attitudinal, and physical environment significantly influences children and young people’s everyday experiences. The synthesis revealed that children with DCD experience bullying and various types of victimisation [61], including, verbal (name-calling, teasing), social (social exclusion, marginalisation), and physical (physical assault) in the school setting. Children with disabilities are at increased risk of bullying in schools in comparison to their typically developing peers [62, 63]. The high prevalence of bullying experienced by children with disabilities is very concerning as strong evidence supports that bullying victimisation in childhood is associated with a wide range of adverse mental health and psychosocial outcomes [64]. Bullying of children because they live with socially devalued identities or characteristics, such as disability, is described as stigma-based bullying and requires bullying interventions specifically focusing on stigma-related factors [65]. Within school environments, greater support to help teachers and peers reconceptualise disability as a social issue may help to challenge notions of marginalisation and discrimination based on socially valued characteristics such as motor proficiency.

The World Health Organisation [66] and childhood disability researchers [67] recognise the importance of contextual factors such as the environment on the health and well-being of individuals. Research has shown that environmental factors (physical, social, and attitudinal) mediate the relationship between child factors and participation outcomes for children and youth with disabilities [68, 69]. For all children, the family represents the most crucial environment, and parents are considered a critical contextual factor in a child’s life [66, 67]. The synthesis findings highlighted the profound influence of the social and attitudinal environmental on children’s experiences within the family context. In line with recent findings regarding the association between family factors and participation of children with a disability [70]; this review found that where parents and siblings understood the child’s strengths and capabilities, participants navigated daily life with greater ease, experienced greater involvement in activities within the home and community environment, and described positive self-perceptions. Conversely, where the home environment was not supportive of the child’s performance and involvement, negative emotions, conflict with parents and siblings, and negative self-perceptions were typical. Our findings support the importance of health service interventions targeted at the family environment. Family-centred approaches that enhance the family’s capacity to understand their child and build competency to resolve challenges may be most beneficial to the child and family [67].

In addition, the school environment was a contextual factor, which significantly influenced opportunities for social inclusion and involvement in activities. Our findings demonstrate the significant influence of teachers and peers on participation and the subsequent positive or negative self-perceptions and school-based experiences of children and young people with DCD. Evidence suggests that teacher awareness and understanding of DCD is poor [71]. The findings of this synthesis highlighted that school sports and physical education were perceived as particularly challenging experiences, which led to incidents of ridicule, self-exclusion, marginalisation, and reluctance to participate due to poor teacher understanding and awareness. As noted by [72], teachers’ knowledge, understanding and confidence about teaching students with a disability is crucial to facilitating inclusive and effective Physical Education (PE). In line with previous research, our results suggest that children and young people’s functional abilities with handwriting, motor, organisation and planning skills influenced their capacity to complete specific activities in school [73]. However, similar to recent findings, involvement in school-based activities was also heavily influenced by the interaction between personal factors such as motivation, interest, preference and environment factors such as the adults, peers, the school structures and routines as well as access to assistive technology and objects [73]. These findings highlight the need for interventions that target teacher collaboration between health professional and educator, in order to build teachers’ knowledge, awareness and capacity to support children and youths with DCD in school. As seen in Canada, Partnering for Change presents an innovate intervention that promotes a model of health and education partnership, which shifts the focus from individualised support to targeting whole school environmental interventions [74]. Society and systems influence the opportunities for inclusion in many aspects of social life such as education, health and community choices [75]. Therefore, further development and evaluation of targeted social/environmental interventions is warranted as they may provide a means to enhance inclusion and reduce social, physical, and attitudinal barriers faced by children and young people with DCD.

Rosenbaum and Gorter [67] provide a novel representation of the ICF concepts, which they define as the F-words (fitness, function, friends, family, fun and future). The results of this study converge with Rosenbaum and Gorter’s [67] representation of the ICF concepts highlighting the importance of friendships, peer social acceptance and fun to support participation in everyday situations. Children and young people with DCD valued friendships and the opportunity for inclusion in social and physical activities with peers across environments. These findings align with previous evidence, which suggest children with disabilities perceive activity experiences as positive, when completed with peers and friends and they are fun [57, 76, 77]. Unsurprisingly, friendships for children with disabilities are associated with greater psychosocial wellbeing [78], social inclusion [79], and healthier identify formation in adolescence [80]. Previous research has revealed that children with a disability face greater risk of social exclusion [81]. However, social inclusion is possible when personal and social resources for the children with a disability are present, including, the ability to make friends, participate in community activities, engage in leisure and play, and have access to quality inclusive practices in the classroom [81]. Our findings emphasise the importance of attending to the social environment so that children and young people with DCD have the opportunity to be involved in meaningful activities that promote social interaction and friendship over time.

In our review, some participants drew on personal resources or accessed supports that facilitated inclusion. Personal resources included a “just do it” attitude, recognising their strengths, accepting and embracing their difference, using humour or sarcasm, and viewing performance and context expectations as a social construct. Our findings corroborate the significance of personal resources such as preferences, interests, and motivations to influence and sustain participation in leisure and school-based activities [73, 76, 82], while supports accessed included, friendships, and support from family and teachers. Positive relationships are considered an important protective factor to help children overcome the risk of social exclusion [81, 83]. The findings of review highlight how the interaction between the personal and environmental factors shaped the lived experiences of children and young people with DCD.

Our integration of findings clearly show that some children and young people experience difficulties because of the mismatch between performance expectations, and their relationships with others including family, teachers and friends. We developed a line of argument and present the experience of children and young people with DCD as reflective of adopting the social relational perspective of the social model of disability [59, 84]. This theoretical perspective provides a lens to contextualise how social and attitudinal restrictions of activity influence the experiences of children and young people with DCD. Children and young people’s perspectives reflect ‘psycho-emotional disablism’ [59]. The social behaviours of others, such as family members, teachers and peers had the potential to undermine their psycho-emotional wellbeing and impose social restrictions of activity. Accordingly, changes are required within the social structures, which create prejudice towards individuals with impairment not to the individual’s psychological adjustment [59], hence in keeping with the social model of disability, interventions that target sigma may be helpful approaches [65].

The review findings corroborate the need for greater attention to the child’s perspective in research and practice, recognising that there are potential discrepancies between children and parents’ perspectives. A recent survey of perceived support for children with DCD in Canadian schools surveyed parents of children with DCD rather than children themselves [85]. Izadi-Najafabadi and colleagues found that the attitudes and actions of teachers, staff, school-related policies and procedures were perceived as adequate by parents [85], whereas the findings of this review reflects that children and young people do not always perceives attitudes and actions of school staff as supportive. Accessing the lived experience of school for children with DCD is important, as children can articulate their experiences and richly inform others on the issues affecting their lives. Another important distinction between the perspective of children and parents is that young people with coordination difficulties, more so than their parents, prioritise friendship as means to promoting self-esteem [86]. The findings of this review corroborate this perspective, friendships were highly regarded by children and young people and influenced their self-perceptions. This review underscores the importance of health professionals exploring the child and young person’s perspectives and priorities for treatment, in conjunction with the parental perspectives in order to build family capacity to manage everyday life situations, line with best practice recommendations [6, 87]. The synthesis findings emphasise the need for professionals to design interventions, which target priority outcomes for friendship, social inclusion and greater understanding and awareness in the school setting, as these are important to children and young people with DCD. Professionals should also explore the child’s strength, and strategies used to navigate everyday life prior to recommending or commencing intervention.

## Strengths and limitations

A major strength of this paper is that it is the first meta-ethnography to focus on the experience of children and young people with DCD. This synthesis addresses the unequal attention conferred to parents versus children’s perspectives. Research examining children’s perspective is essential if we are to improve the lives of children [88].

A strength of this paper is the robust conduct of the review; the authors adhered to the eMERGe and PRIMSA guidelines. However, a limitation of this review was the quality of included studies varied. Furthermore, the phenomenon of interest examined across the included studies was diverse, which resulted in a focus on certain aspects or dimensions of children’s and young people’s experience in some papers. For example, Hessell and colleagues’ examination of the experience of a gymnastics club for children with DCD [58], or Armitage and colleagues’ investigation of children’s perceptions and experiences of occupational therapy intervention [49].

## Implications for practice

The findings of the synthesis point to the need for health professionals working with this population to consider and address the social and attitudinal environments in which children and young people participate and live, but especially the school environment. It is important that interventions, which support teacher knowledge, awareness and understanding of DCD, be developed. Such interventions are one element of promoting a positive social and attitudinal school environment for children with DCD. For example, increasing teacher awareness and recognising the challenges associated with fatigue, fear of injury or poor performance in front of peers during physical education classes may help to support involvement.

Health service provision should adopt a family-centred approach that enhances the capacity of the family to support engagement and performance in activities and everyday situations. Augmenting parents understanding of DCD may be helpful in addressing potential negative child self-perceptions arising from parental expectations. Finally, children and young people described numerous successful strategies to deal with challenging experiences, victimisation, and exclusion. Thus, greater attention is required by professionals to identify children’s capabilities and strategies, which may be leveraged to address challenging social interactions and navigate daily life activities.

### Future research

Most qualitative studies included in this review explored lived experiences of children with DCD in general. We identified no qualitative studies focusing exclusively on the experiences of friendship or romantic relationships, education, or transition to work or vocational roles or parenting with DCD for example, and these topics could be the focus of future work. Future research needs to explore children’s experiences of friendships and social inclusion. Furthermore, research needs to broaden the focus of examination; and explore how children and young people with DCD deal with life situations and specific activities. This review highlighted that children and young people implement many strategies to support their involvement in everyday life. Children’s experiences and coping skills needs to be examined in-depth to inform future intervention development and help to guide recommendations for practice. Moreover, future studies should be designed with Public and Patient Involvement from the outset to ensure that the concerns and priorities of children and young people with DCD are addressed in research conducted. Guidelines on co-producing research with young people exist and can provide a framework to support the inclusion of children and young people’s voices throughout the research process [89, 90].

## Conclusion

This paper exemplifies the value of qualitative research to inform research, policy, and practice. Our synthesis of fifteen papers produced three interrelated themes; a) ‘It’s harder than it should be’: Navigating daily activities, b) Fitting in, and c) ‘So what? I drop things’: Strategies and supports to mitigate challenges. Children with DCD describe a mismatch between their abilities and performance norms for daily activities that could led to a cascade of negative consequences including negative self-appraisal, bullying and exclusion. In the face of these difficulties, children with DCD described friendships as a forum for fun, acceptance and protective against exclusion and bullying and they described a range of creative and successful strategies they enacted and supports they accessed.

## Supporting information

S1 File Search strategy MEDLINE. (DOCX)

S2 File PRISMA 2009 checklist. (DOC)

S3 File Inclusion & exclusion criteria. (DOCX)
