# Supplementary material for: Children and young people’s experiences of living with developmental coordination disorder/dyspraxia: A systematic review and meta-ethnography of qualitative research
Source: PLoS One. 2021 Mar 4;16(3):e0245738. doi: 10.1371/journal.pone.0245738 (PMC7932121; doi:10.1371/journal.pone.0245738)
Supplement: S1 File — (DOCX) [file pone.0245738.s001.docx]

| **S1 File. Search Strategy MEDLINE** | |
| --- | --- |
| **S1** | Motor Skills Disorder* OR developmental coordination disorder OR clumsiness OR clumsy OR in-coordination OR dys-coordination OR minimal brain dysfunction OR minor neurological dysfunction OR motor delay disorder OR perceptual-motor impairment OR motor coordination difficulties OR motor learning difficulties OR mild motor problems OR non-verbal learning disability OR non-verbal learning disorder OR non-verbal learning dysfunction OR motor coordination problems OR sensorimotor difficulties OR sensory integrative dysfunction OR physical awkwardness OR physically awkward OR psychomotor disorders OR motor control and perception OR developmental dyspraxia OR perceptual motor dysfunction OR minimal cerebral dysfunction |
| **S2** | qualitative OR experience* OR perception* OR perspective* OR case stud* OR interview* OR focus group* OR mixed methods OR participant observation OR transcript* OR ethnograph* OR phenomenol* OR grounded theor* OR grounded-theor* OR purposive sample OR lived experience* OR narrative* OR life experience* OR life stor* OR action research OR observational method OR thematic analysis OR narrative analysis OR field stud* OR field-notes OR videorecording |
| **S3** | child OR children OR adolescent OR teen OR teenager OR youth OR young person OR young adult |
| **S4** | S1 AND S2 AND S3 |
