# Supplementary material for: Children and young people’s experiences of living with developmental coordination disorder/dyspraxia: A systematic review and meta-ethnography of qualitative research
Source: PLoS One. 2021 Mar 4;16(3):e0245738. doi: 10.1371/journal.pone.0245738 (PMC7932121; doi:10.1371/journal.pone.0245738)
Supplement: S3 File — (DOCX) [file pone.0245738.s003.docx]

| S3 File. Inclusion & Exclusion criteria | | |
| --- | --- | --- |
|  | **Inclusion Criteria** | **Exclusion criteria** |
| **Sample** | Children aged five to eighteen years with a diagnosis of DCD or probable DCD.  Participants with DCD and a co-occurring specific learning difficulty or neurodevelopmental diagnosis such as ADHD will be included as co-occurrence is very common (Blank et al., 2019).  Participants must meet the Diagnostic and Statistical Manual of Mental Disorders 5^th^ Edition (DSM-V) criteria for DCD.  Where children and young people are described as having probable DCD, the authors must outline how each criterion of the DSM-V was fulfilled:   1. motor impairment scores below the 15^th^ percentile on a standardised motor test; 2. describe how the participants’ activities of daily living are affected as a result of the motor skills difficulties 3. explain the participants cognitive ability and confirm that it is within the normal intellectual ranges 4. indicate that no underlying medical condition is reported by parents, guardians, teachers or health professionals.   Studies examining parental and child experiences will be included, but it must be possible to extract data on the child and young person views and experiences of living with DCD. | Children younger than five years will be excluded as a diagnosis of DCD is not confirmed below five years of age (Blank et al., 2019).  Studies that include a sample of children and young people with a different diagnosis will be excluded if it is not possible to extract the views and experiences of children and young people with DCD within such studies.  Studies examining the opinions and experiences of parents of children with DCD will be excluded. |
| **Phenomenon of interest** | Children and young people who describe their views, opinions and experiences of living with DCD. |  |
| **Design** | Qualitative or mixed-methods studies reporting primary qualitative data (e.g., data collected through qualitative methods such as interviews, focus groups, or participant observation etc.) | Where the qualitative data from the child cannot be identified, such as summaries or aggregated data of parent and child experiences, these papers will be excluded. |
| **Evaluation** | Qualitative analysis of experiences, feelings,  views, opinions, and experiences of living with DCD. All settings such as school, home, community, etc. will be included. | Studies where a method of qualitative analysis is not described. |
| **Research type** | Peer-reviewed journal articles and thesis.  Full text available in English  Published between No date limit- 2019 | Systematic reviews, protocols, theoretical work, editorials, opinion pieces, dissertations, grey literature. |
